# The cerebral hemodynamic response to phonetic changes of speech in preterm and term infants: The impact of postmenstrual age

**DOI:** 10.1016/j.nicl.2018.05.005

**Published:** 2018-05-15

**Authors:** Takeshi Arimitsu, Yasuyo Minagawa, Tatsuhiko Yagihashi, Mariko O. Uchida, Atsuko Matsuzaki, Kazushige Ikeda, Takao Takahashi

**Affiliations:** a Department of Pediatrics, Keio University School of Medicine, Shinjuku, Tokyo 160-8582, Japan; b Department of Psychology, Faculty of Letters, Keio University, Kohoku-ku, Yokohama 223-8521, Japan; c Graduate School of Human Relations, Keio University, Minato-ku, Tokyo 108-8345, Japan

**Keywords:** BPD, bronchopulmonary dysplasia, NEC, necrotizing enterocolitis, MMN, mismatch negativity, PMA, postmenstrual age, fNIRS, functional near-infrared spectroscopy, Oxy, oxygenated, Deoxy, deoxygenated, HRF, hemodynamic response function, GA, gestational age, PNA, postnatal age, SOA, stimulus onset asynchrony, ROI, region of interest, BOLD, blood oxygenation level dependent, IQR, interquartile range, Near-infrared spectroscopy, Preterm infants, Laterality, Speech perception

## Abstract

Higher brain dysfunction, such as language delay, is a major concern among preterm infants. Cerebral substrates of cognitive development in preterm infants remain elusive, partly because of limited methods. The present study focuses on hemodynamic response patterns for brain function by using near-infrared spectroscopy. Specifically, the study investigates gestational differences in the hemodynamic response pattern evoked in response to phonetic changes of speech and cerebral hemispheric specialization of the auditory area in preterm infants (*n* = 60) and term infants (*n* = 20). Eighty neonates born between 26 and 41 weeks of gestational age (GA) were tested from 33 to 41 weeks of postmenstrual age (PMA). We analyzed the hemodynamic response pattern to phonemic and prosodic contrasts for multiple channels on temporal regions and the laterality index of the auditory area. Preterm infants younger than 39 weeks of PMA showed significantly atypical hemodynamic patterns, with an inverted response shape. Partial correlation analysis of the typicality score of hemodynamic response revealed a significant positive correlation with PMA. The laterality index of preterm infants from 39 weeks of PMA demonstrated a tendency rightward dominance for prosodic changes similar to term infants. We provide new evidence that alterations in hemodynamic regulation and the functional system for phonemic and prosodic processing in preterm infants catch up by their projected due dates.

## Highlights

• Hemodynamic responses to speech were examined in 80 preterm and term neonates.
• fNIRS measured hemispheric specialization to phonemic and prosodic contrasts.
• Some preterm infants showed atypical hemodynamics with an inverted response shape.
• Postmenstrual age was related to hemodynamic patterns and functional lateralization.
• Functional hemodynamic regulation of preterm infants caught up by the expected dates.

## 1 Introduction

Major disabilities in preterm infants are becoming less frequent as medical technologies advance. However, higher rates of brain dysfunction in such infants compared with term infants remain an issue (Mwaniki et al., 2012). Even if preterm infants do not present with major central nervous system disorders or other significant complications (e.g., grade 2 to 4 intraventricular hemorrhage, periventricular leukomalacia, bronchopulmonary dysplasia, BPD or necrotizing enterocolitis, NEC) at discharge from hospital, higher brain dysfunctions may appear during development (Luu et al., 2009; Aarnoudse-Moens et al., 2009). Such dysfunctions in the cognitive system can be examined by neuroimaging of the brain function and brain anatomy of infants. However, assessment of the hemodynamic response function to cognitive processing could also reveal some aspects of physiological traits of higher brain functions.

Higher brain dysfunction should be identified in the early stages of development to enable early intervention. However, methods for early detection among preterm infants have not yet been established (Mento and Bisiacchi, 2012). Development of such methods is hindered by the lack of research into the neuronal substrates associated with early speech perception in preterm infants, even though this is one of the most important higher brain functions (Mento and Bisiacchi, 2012; Tusor et al., 2014). Studying these processes in preterm infants will provide insights into early developmental milestones and the relationship between brain maturity and function.

EEG studies of term and preterm infants have provided evidence of the neuronal processes underlying early speech perception, with a particular focus on auditory-evoked potentials called mismatch negativity (MMN). For instance, prematurely born infants exhibited MMN to phonemic contrasts of /y/ and /i/, suggesting they had the ability to discriminate between them (Cheour-Luhtanen et al., 1996). However, preterm infants tend to show smaller MMN amplitude than do age-matched full-term infants in their first year of life (Alho et al., 1990). Likewise, recent EEG studies have reported that neural maturation, reflected by postmenstrual age (PMA), has a large impact on speech discrimination (Pena et al., 2010; Bisiacchi et al., 2009). EEG studies have also revealed the developmental course of language-specific phonemic processing, showing developmental changes to produce stronger MMN to native phonemic contrast (Cheour et al., 1998; Dehaene-Lambertz and Gliga, 2004). Moreover, the MMN index in 7.5-month-olds was shown to predict language development at 2 years old (Kuhl et al., 2008).

Although EEG studies have provided accumulating evidence on the functional neurodevelopment as reviewed above, functional near-infrared spectroscopy (fNIRS) is an emerging neuroimaging technique that can strengthen the role of EEG study in the understanding of brain development. This technique has better spatial resolution and so can increase our knowledge of the cerebral substrates for receptive language in preterm infants, particularly hemispheric specialization of specific brain regions. Although the temporal resolution of fNIRS is lower than that of EEG, fNIRS uses an infant-friendly headset without any paste or gel and is a more portable system, which has better temporal resolution (10 Hz) than fMRI. fNIRS measures neuronal activity reflected in changes in concentrations of oxygenated (oxy-) Hb and deoxygenated (deoxy-) Hb and it has been used to identify various neurocognitive developmental processes in infants (Minagawa-Kawai et al., 2011; Gervain et al., 2011; Minagawa-Kawai et al., 2008). Such findings have elucidated the cerebral responses of infants to two spoken language components: phonemes and prosody (Sato et al., 2003; Arimitsu et al., 2011). Phonemic structures (e.g., vowels and consonants) tend to be processed predominantly in the left temporal area, while prosody (e.g., intonations or rhythms) activates the right side more in both children and adults. This functional hemispheric specialization is called functional cerebral laterality of the auditory cortices and it facilitates efficient processing in the cerebral cortex (Minagawa-Kawai et al., 2011; Sato et al., 2003; Arimitsu et al., 2011).

Although language comprehension involves various processes, the perceptual analysis of phonemes and prosody is a crucial initial step for language acquisition in the first year of life. Indeed, perceptual analysis of phonemes deeply affects an infant's learning of their native language. Likewise, prosody is important, because it provides various cues, such as pitch changes, intensity, and rhythmic structures—all of which facilitate the infant's speech acquisition, as exemplified by infant-directed speech offering rich prosodic cues.

Apart from the investigations of cognitive functions stated above, fNIRS is also one of the significant tools to examine hemodynamic physiology in infants as well as in adults. Typical hemodynamic activity of the adult cerebral cortex is characterized by an increase in oxy-Hb and a slight decrease in deoxy-Hb. This is known as the hemodynamic response function (HRF) (Boynton et al., 1996; Friston et al., 2000). However, the development of the HRF pattern in the human brain remained unclear, and some fNIRS studies reported an inverted HRF pattern for young infants in response to perceptual stimuli, whereas some studies did not (Minagawa-Kawai et al., 2008; Sato et al., 2003; Arimitsu et al., 2011). This has been a controversial issue, and evidence of its presence in preterm infants is particularly sparse, partly because the fNIRS investigation of HRF is a relatively new subject. Notably, a recent fNIRS study reported developmental changes in phase differences of oxy- and deoxy-Hb in preterm infants (Watanabe et al., 2017). Although such developmental changes in neurovascular regulation are very intriguing, these results come only from resting-state measurements; HRF patterns during perceptual or cognitive processing should also be investigated.

Consequently, the present study attempts to determine developmental differences in the HRF in response to different functional speech stimuli in preterm and term infants at each PMA. For stimuli, we employed well-established instances of linguistic contrast (phonemic and prosodic contrasts) that are crucial for early language development, as mentioned above. In this study, we specifically examined two aspects of the brain response in neonates: (a) the hemodynamic response pattern of oxygenated (oxy)-Hb changes and (b) functional hemispheric specialization in the temporal cortices. We chiefly focused on PMA, also taking gestational age (GA), post-natal age (PNA), and birth weight into consideration, because HRF is closely related to the physiological development of neonates' vascular systems, which may continuously develop before and after birth. Furthermore, as stated above, the EEG literature on speech perception has reported a significant role of neural maturation corresponding to PMA.

Previous fNIRS studies have also reported increased functional hemispheric specialization during development in the first year of life, reporting developmental changes in laterality but with a normal HRF in every age group. However, no study so far has attempted to examine functional cerebral laterality and HRF regulations in preterm infants (Minagawa-Kawai et al., 2011). We therefore explore whether preterm infants show functional cerebral laterality similar to that reported in term infants.

## 2 Methods

### 2.1 Participants

The parents of participants were approached for consent and enrollment between 2010 and 2012. The study included 20 term and 60 preterm neonates, who were all from monolingual Japanese families. Participants were divided into four groups according to their PMA at time of examination. Demographic data for each group are shown in Table 1. An additional 16 neonates were excluded because of noise due to motion artifacts and/or loose probe attachments.

**Table 1:** Characteristics of participating infants grouped by PMA at time of examination. IQR stands for interquartile range.

	Preterm infants			Term infants
Group of CGA at the examination	33–35 weeks (*n* = 27)	36–38 weeks (*n* = 17)	39–41 weeks (*n* = 16)	37–41 weeks (*n* = 20)
Male, n (%)	14 (51.9)	8 (47.1)	7 (43.8)	9 (45.0)
Age at the examination, days, median (IQR)	16 (12–27.5)	17 (9–29)	49 (43.75–56)	4 (3.75–6.25)
GA at birth, weeks, median (IQR)	32 (30−33)	33 (32–35)	32 (31–34)	38 (37–39)
Birth weight, g, median (IQR)	1668 (1313–1898)	1733 (1421–1834)	1614 (1453–1846)	2798 (2705–3029)
Apgar score at 1 min, median (IQR)	7 (5.5–8)	8 (6–8)	7 (4.75–8)	8 (8–9)
Apgar score at 5 min, median (IQR)	8 (7.5–9)	9 (8–9)	8 (8–9)	9 (9–9)
Weight at the examination, g, median (IQR)	1886 (1773–2046)	1940 (1850–2228)	3007 (2980–3302)	2735 (2638–2948)

The GA was determined by an obstetrician using data including the last menstrual period, the first accurate ultrasound examination, and assistive reproductive technology. We excluded infants with chromosomal or congenital anomalies including congenital heart anomalies, grade 2 to 4 intraventricular hemorrhage, periventricular leukomalacia, moderate and severe BPD defined as per the National Institutes of Health criteria, NEC, deafness diagnosed by automated auditory brainstem response and those who were medically unstable (Jobe and Bancalari, 2001). Ductus arteriosus was clinically closed at the time of examination in infants whose birth weight was >1500 g. For infants whose birth weight was <1500 g, the closure of ductus arteriosus was confirmed by echocardiography before the fNIRS measurement. This study was conducted at the Keio University Hospital (Tokyo, Japan). The institutional review boards of the hospital approved all protocols related to the study, and informed consent was obtained from the parents of all participating infants.

### 2.2 Stimuli and conditions

For stimulus words, we used three different forms of one Japanese verb /itta/, /itte/, and /itta?/. These sounds were synthesized from speech signals produced by a male adult as described elsewhere (Imaizumi et al., 1998). The two main experimental conditions were the phonemic contrast (/itta/ vs. /itte/), which differed in the final vowel, and the prosodic contrast (/itta/ and /itta?/), which differed in pitch contours. We employed a block design where the target block for these two conditions was alternately presented against an identical baseline block. Specifically, participants received a baseline block where the stimulus /itta/ was repeated with 1 s of stimulus onset asynchrony (SOA) for a total of 15 s. They then received another 15 s of the target block for either the phonemic or prosodic condition. Under the phonemic target block, /itta/ and /itte/ were presented in a pseudo-random order at 1 s of SOA. Similarly, the prosodic condition comprised a serial presentation of /itta/ and /itta?/ in a random order. The two blocks (baseline and target blocks) under each condition were alternated at least seven times per condition.

### 2.3 Procedure

**Fig. 1:**
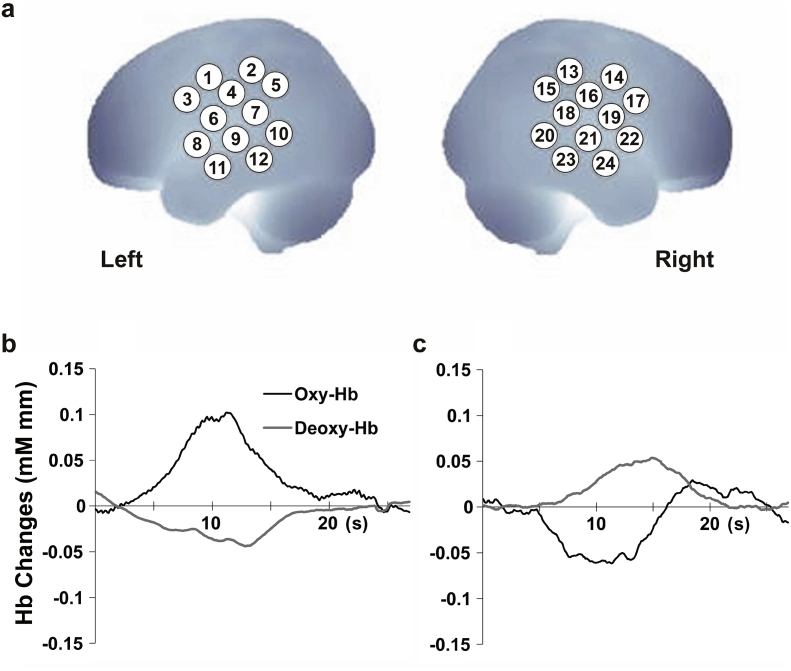
HRF patterns in the temporal regions. (a) Location of 12 channels for each hemisphere. Each channel is represented by a number. Channels 1 to 12 are located on the left hemisphere (left) and channels 13 to 24 are located on the right hemisphere (right). (b, c) Representative examples of time courses of Hb changes. The typical HRF pattern is characterized by an increase in oxy-Hb and a slight decrease in deoxy-Hb (b), while the inverted pattern is characterized by a decrease in oxy-Hb (c).

The auditory-evoked responses in the bilateral temporal area and part of the frontal regions were recorded using fNIRS (ETG 4000, Hitachi Medical Corporation, Tokyo, Japan). A silicon pad with five incident and four detection probes, arranged in a 3 × 3 square lattice with a separation of 20 mm, was placed laterally on each side of the infant's head (Fig. 1a). Each pad comprising 12 channels was attached to the head so that the center detector probe at the bottom of the horizontal probe line corresponded with the T3 or T4 position in the international 10/20 system as described elsewhere (Boynton et al., 1996). Over these two probe pads, thin plastic plates were inserted for better attachment to the head; these pads and plates were held in place by elastic bands. Average infant head circumference was 30.8 cm (SD = 2.1) for the youngest group and 33.8 cm (SD = 0.8) for the oldest group, and this was statistically different (*P* < 0.01). Taking 4.0 cm of the probe pad into consideration, this creates a difference of 1.1% coverage of the measurement area for each temporal pad. Although this indicates a slightly larger measurement area for younger infants, this difference is negligible considering the spatial resolution of fNIRS. The infants were tested when asleep and received stimulation at amplitudes of approximately 67 dB via two speakers, which were positioned about 45 cm away from the participant.

### 2.4 Data analysis

First, we analyzed each channel separately to assess whether the oxy-Hb response increased or decreased with the shape of a typical HRF. We aimed to examine the general hemodynamic time course, so we analyzed all channels used. We focused on the oxy-Hb response because this general indicator was greater than the deoxy-Hb response (Minagawa-Kawai et al., 2007; Lloyd-Fox et al., 2010). Next, a region of interest (ROI) in the temporal auditory area was tested to examine functional hemispheric specialization (Fig. 1a). For spatial estimation of the channel location in the brain, we employed the virtual registration method to map fNIRS data onto the MNI standard brain space (Tsuzuki et al., 2007). Oxy- and deoxy-Hb concentrations were calculated from the absorption of 695 and 830 nm laser beams sampled at 10 Hz and smoothed with a 1-second moving average.

We used the same methods to those in our previous study for data preprocessing, including artifact rejection, data blocking, and detrending (Arimitsu et al., 2011). The time courses of Hb concentration changes in the analysis blocks were averaged more than four times for each of the stimulus conditions. To determine whether the obtained time course of the Hb changes fits with the typical HRF pattern or an atypical reversed one, the correlation between the canonical HRF model (Boynton et al., 1996; Friston et al., 2000) and the oxy-Hb data was calculated for each channel for each infant. The canonical HRF model uses a Gaussian smoothing kernel which represents the typical HRF pattern and is regularly employed in fMRI analysis (Boynton et al., 1996; Friston et al., 2000). A positive correlation coefficient suggests an increased pattern similar to the typical HRF model, while a negative value represents a decreased pattern, indicating an atypical HRF pattern. These values are then transformed to Fisher's *Z*-score, which we denoted the HRF-typicality score. From these data, the average HRF-typicality score for 24 channels was obtained for the phonemic and prosodic conditions in each infant.

**Supplementary Fig. S1:**
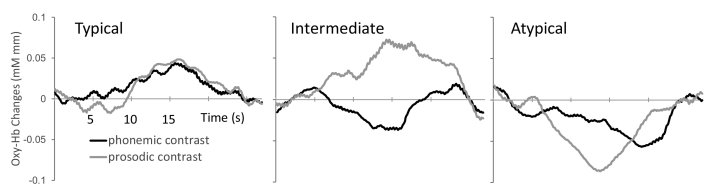
Some examples of HRF patterns different in categorical labels (i.e. Typical, Intermediate, Atypical). Positive responses (positive HRF typicality score) for both conditions are categorized to “Typical”. Positive and negative responses for one of the two conditions are categorized to “Intermediate”. Negative responses for both of the conditions are categorized to “Atypical”.

We categorized the participants into three types: (1) “typical” where the oxy-Hb change showed an increased pattern (positive HRF-typicality score) for both phonemic and prosodic conditions, (2) “intermediate” where oxy-Hb increased for only one condition, and (3) “atypical” where oxy-Hb decreased (negative HRF-typicality score) for both conditions (Fig. 1b and Supplemental Fig. S1). In addition to this categorical assessment, we performed ANOVAs to investigate the HRF-typicality scores. For this analysis, the dependent variables were the averaged HRF-typicality scores of phonemic and prosodic conditions. Finally, we performed regression analyses to examine the relationship between the HRF-typicality score and PMA, controlling for PNA at examination, birth weight and GA.

Next, to examine functional hemispheric specialization, we followed the same method for calculating the laterality index as described elsewhere (Sato et al., 2003; Arimitsu et al., 2011; Minagawa-Kawai et al., 2007; Sato et al., 2007). This involved first defining an ROI in the auditory area at channels 6, 8, 9, and 11 on the left hemisphere and channels 19, 21, 22, and 24 on the right (Fig. 1a). These channels were chosen because their locations likely incorporate the auditory areas, according to the virtual registration method (Tsuzuki et al., 2007). For each participant, we selected one channel that showed the maximum oxy-Hb response within the auditory area. We employed this method as it has been used in previous studies that have successfully elicited hemispheric lateralization (13, 16, 17, 22, 25,). We used the peak oxy-Hb value 5–15 s after stimulus onset for channel selection. For negative HRF-typicality scores, we took the negative peak. The laterality index was calculated using these values and the formula (L − R)/(L + R), where L and R are peak values on the left and right sides, respectively.

## 3 Results

### 3.1 Impact of PMA on development of HRF patterns

In response to speech stimulation, preterm infants showed variable patterns of auditory-evoked hemodynamic changes in the measured channels (Fig. 1a–c). Term infants generally demonstrated a HRF typically seen in adults, with an increase in oxy-Hb and a slight decrease in deoxy-Hb in the temporal area in response to speech stimulation (Minagawa-Kawai et al., 2007; Lloyd-Fox et al., 2010) (Fig. 1b). However, preterm infants demonstrated variable Hb patterns, with either a similar pattern to term infants (Fig. 1b; typical Hb pattern) or an inverted pattern with decreased oxy-Hb (Fig. 1c; atypical Hb pattern). Regardless of the direction of HRF, all the neonates showed consistent responses to the target stimuli compared with the baseline stimuli.

**Fig. 2:**
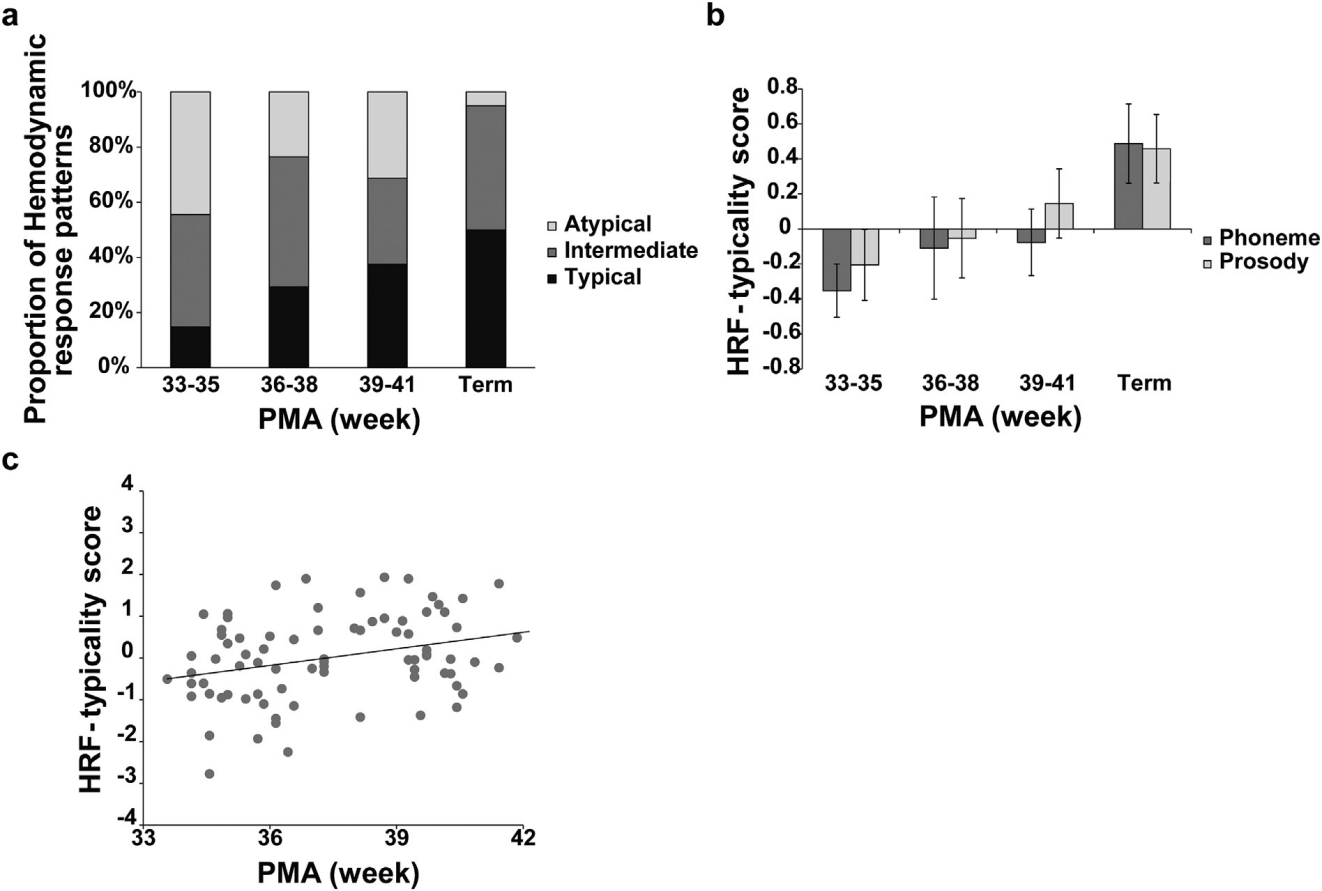
The HRF pattern according to PMA. (a) The proportion of HRF patterns for each group. The three types of categories are: “typical” showing the normal HRF for two conditions, “intermediate” showing a normal HRF for one condition, and “atypical” showing an inverted HRF for two conditions. (b) HRF-typicality scores according to stimuli and PMA group. (c) Correlation between HRF-typicality scores and PMA (R = 0.30, *P* = 0.006).

To objectively assess the typicality of HRF patterns, we calculated the fit between the data and the canonical HRF model. Then we created three categories using HRF-typicality scores from the average data of 24 channels. If the score was positive for both of stimuli conditions, it was grouped as “typical”. If one of the scores was negative, it was categorized as “intermediate” (see Methods for more detail). Fig. 2a shows the proportions of HRF patterns for each PMA group. The proportion of infants with a “typical” HRF increased with PMA. Conversely, the proportion of infants with an “atypical” HRF decreased with PMA. A similar pattern was also observed in HRF-typicality scores depending on stimulus condition (Fig. 2b). Thus, positive HRF-typicality scores gradually increased as PMA increased.

To confirm this pattern, two-way ANOVAs were conducted on the HRF-typicality scores, with PMA as a between-subject factor and the stimuli (prosody and phoneme) as a within-subject factor. The results indicated a main effect of PMA [F(3,76) = 3.86, *P* = 0.01, *η*^2^ = 0.15]. There was no significant effect of stimulus type [F(1,76) = 0.50, *P* = 0.48, *η*^2^ = 0.00] and no significant interaction between PMA and stimulus type [F(3,76) = 0.15, *P* = 0.92, *η*^2^ = 0.00]. Homogeneity of variance among different age groups was confirmed [F(3,156) = 1.33, *P* = 0.26] using Levene's test.

Because PMA had a significant effect on HRF-typicality scores, we further analyzed the differences between each PMA group using a post-hoc test (Ryan's method). Significant differences were observed between the term group and both the 33–35 weeks of PMA group (t (76) = 3.59, *P* < 0.001, d = 0.82) and the 36–38 weeks of PMA group (t (76) = 2.37, *P* = 0.02, d = 0.57). No significant differences were found between the term infants and the group of infants at 39–41 weeks of PMA. The results demonstrated that regardless of stimulus type, the HRF pattern for speech stimulation in preterm infants developed with PMA, becoming more similar to that in term infants from 39 weeks of PMA.

### 3.2 Correlation between PMA and HRF patterns

Having demonstrated the significant effect of PMA on HRF patterns, we evaluated the correlation between PMA and HRF-typicality scores. Simple correlation analysis demonstrated a significant relationship between PMA and HRF-typicality scores (*R* = 0.304, *P* = 0.006; Fig. 2c). To exclude possible effects on HRF-typicality scores of factors other than PMA, we conducted a partial correlation controlling for PNA at examination, birth weight and GA. PMA still showed a significant correlation with HRF-typicality scores after controlling for PNA at examination (*R* = 0.32, *P* = 0.002) and birth weight (*R* = 0.206, *P* = 0.034). The correlation was marginally significant after controlling for GA (*R* = 0.175, *P* = 0.062). This indicates that PMA can well explain typicality scores in the present dataset, but there is also a moderate influence of GA.

### 3.3 PMA and functional hemispheric specialization in speech perception

**Fig. 3:**
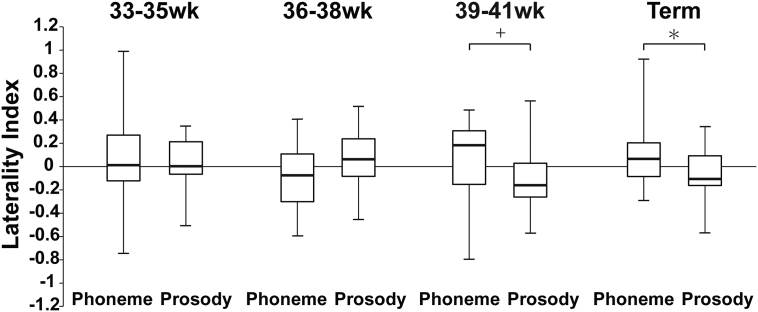
Laterality indices for phonemic and prosodic conditions according to PMA group. A positive value means left-dominance, whereas a negative value means right-dominance. * = *P* < 0.05.

The laterality index was calculated for each PMA group. As observed in Fig. 3, term infants show a tendency to right-dominant responses to prosodic contrasts, as most of the laterality indices (LI) are below zero in contrast to the higher LI for the phonemic contrast. This tendency to a right-sided reduction is also seen in the oldest PMA group. Two-way ANOVAs comparing LI indices, with PMA and stimulus condition as independent variables, indicated a marginally significant interaction between PMA and the stimuli [F(2,77) = 2.71, *P* = 0.050, *η*^2^ = 0.01]. A simple main effect test further revealed that this interaction was due to the group of term infants [F(1,76) = 5.03, *P* = 0.027] and those at 39–41 weeks of PMA [F(1,76) = 3.95, *P* = 0.050]. These two groups showed lower LI for the prosodic contrast. Namely, these groups showed lower LI in the prosodic contrast than in the phonemic contrast.

## 4 Discussion

Although the prognosis of preterm infants has improved, understanding of the effects of preterm birth on neurodevelopment of various language functions remains elusive despite their significance. In this study, we examined functional hemodynamic regulation using linguistic stimuli and demonstrated that the HRF pattern for phonemic and prosodic stimulation in preterm infants developed with PMA, becoming similar to that in term infants after 39 weeks of PMA. After 39 weeks of PMA, the laterality index for prosody processing was lower than that for phoneme processing. In term infants, prosodic structure was processed predominantly in the right auditory area. Together, these results indicate that preterm infants begin to demonstrate right-dominant functional cerebral laterality for prosody after 39 weeks of PMA, such that they come to resemble term infants. The present study succeeded in using fNIRS to capture developmental differences in auditory-evoked neuronal activity represented by the HRF and functional hemispheric specialization of auditory areas that process speech contrasts. Consistent with previous studies showing a discriminative response to phonemic differences in preterm infants, even at 32 weeks of PMA, we also observed differences in the hemodynamic response to phonemic and prosodic differences (Cheour-Luhtanen et al., 1996; Mahmoudzadeh et al., 2013). Our results further suggest that in preterm infants both the cortical HRF and functional hemispheric specialization in temporal regions related to speech stimulation normalize to resemble term infants at 39 weeks of PMA. This was demonstrated in a relatively large sample of preterm and term infants. It is possible that these fNIRS markers represent a milestone of early language development.

In this study, before 36 weeks of PMA, approximately half of the infants showed an atypical HRF pattern for both phonemic and prosodic auditory stimulation. Only about 10% of infants showed a typical HRF pattern. Indeed, the average HRF-typicality score for term infants was significantly different from infants at 33–35 and 36–38 weeks of PMA. The average HRF-typicality score in preterm infants gradually increased in proportion to PMA, until 39 weeks of PMA, at which point they resembled term infants.

What neurophysiological processes does the atypical Hb response reflect in preterm infants? The physiological mechanisms underlying the reversed Hb response of preterm infants remain unclear. However, it could possibly be explained by immature neurovascular and/or metabolic systems in the cerebral cortex. Specifically, immature functioning of the synaptic structure with less myelination might result in inefficient energy use requiring more oxy-Hb (Kozberg et al., 2013). Furthermore, because of the immaturity of the arteriole vessels and capillaries, blood flow may not be sufficient in response to brain activity in preterm neonates. The resulting lack of oxygenation is reflected in the inverted HRF. While it is well-known that synaptic development rapidly occurs during the 6 months after birth, capillary formation starts between term to 3 months of age (Norman and O'Kusky, 1986; Huttenlocher, 1990). A recent fNIRS study examining phase synchrony of oxy-Hb and deoxy-Hb (Watanabe et al., 2017) further revealed rapid changes in neurovascular regulation from 34 PMA to term infants, which is relatively consistent with our results. We therefore speculate that brain maturity in terms of both synaptogenesis and angiogenesis in preterm and term neonates is a possible factor influencing the hemodynamic response pattern. In particular, maturity of the neurovasculature of the perisylvian area may specifically affect language processing.

Although we demonstrated clear developmental differences in the HRF in preterm infants, the HRF in infants has been a controversial and critical issue in the neuroimaging literature. In contrast to the oxy-Hb increase typically observed in adults, young infants sometimes show an inverted (i.e., decreased) pattern of oxy-Hb or a negative fMRI blood oxygenation level dependent (BOLD) response, as observed in our study (Csibra et al., 2004; Kusaka et al., 2004; Born et al., 1996). However, results have been inconsistent and other studies have reported typical Hb patterns even in neonates (Taga et al., 2003). This diversity may partly be explained by differences in the cognitive stimuli or task given to the infants and their state of wakefulness (Kusaka et al., 2004; Meek et al., 1998). A recent fNIRS study (Mahmoudzadeh et al., 2013) reported that preterm neonates approximately 31 weeks of PMA responded to phonemic differences. These results are in line with our own data showing preterm infants' sensitivity to phonemic differences. However, in contrast to our results, the authors observed a typical HRF for very premature infants. This discrepancy can be explained by differences in the task, specifically the perceptual component engaged by the task. Specifically, our task paradigm used speech stimulation even for the baseline time period, which differed from their study using silence (Mahmoudzadeh et al., 2013). Our paradigm made it possible to elucidate a pure phonetic component related to phonemic/prosodic differences by excluding sensory components that may be due to whether sound is present or not. Therefore, it appears that maturity of HRF-typicality may also be dependent on the level of neurocognitive processing. Our paradigm, using sounds at baseline, may have imposed a greater neuronal load than a silent baseline, thus requiring more neurovascular activity in the neonates.

Another crucial factor influencing Hb patterns is the targeted brain region. While an inverted pattern is frequently observed in the occipital area, this pattern has not often been reported in the temporal area (Arimitsu et al., 2011; Gervain et al., 2008). Different brain regions follow different developmental pathways, and it appears that development may be slower in the occipital cortex than in the temporal cortex (Lin et al., 2013). Such region-dependent development should be further explored in future studies. In this study, we presented the same auditory stimulation to infants and measured the same perisylvian area in infants at different PMAs. Using this paradigm, we observed that preterm infants tended to show an inverted HRF in the temporal area, and that a typical HRF developed gradually as PMA increased.

We employed phonemic and prosodic speech contrasts, which have been shown to elicit Hb changes in previous studies of functional cerebral laterality (Sato et al., 2003; Arimitsu et al., 2011). These studies suggest that functional hemispheric specialization in auditory areas emerges at term birth and 11 months of age for phonemic and prosodic contrasts, respectively. However, no study has examined this developmental process in preterm infants (Sato et al., 2003; Arimitsu et al., 2011). Our results indicate that rightward dominance appears at 39–41 weeks of PMA in preterm infants such that they resemble term infants. This is consistent with recent studies comparing speech perception in term and preterm infants (Pena et al., 2010; Pena et al., 2012). Earlier maturation of the right hemisphere in both term and preterm infants can partly be explained by the right-dominance of cerebral hemodynamic and oxygen metabolism. Specifically, the level of cerebral hemoglobin oxygenation, the blood volume, and the metabolic rate of oxygen are reported to be greater in the right hemisphere particularly in temporal and occipital areas (Lin et al., 2013).

The tendency to right-dominance for prosodic processing in preterm infants from 39 weeks of PMA suggests that they could process prosodic variation as efficiently as term infants. This also implies that preterm infants, at their projected due dates, possess adequate perceptual functioning to process infant-directed speech by their mother, which is characterized by rich prosodic information, such as intonation and amplitude. Because stimulation with infant-directed speech is important for language development, our results may also indicate that language function in preterm infants can develop as well as that in term infants by hearing infant-directed speech. This may support the efficacy of early intervention for language delay (Zimmerman et al., 2009; Caskey et al., 2011; Spittle et al., 2012). Preterm infants after 39 weeks of PMA have a similar cerebral response to speech as term infants. As such, they can at least absorb phonemic and prosodic components, which are crucial for language development, as well as term infants can. Thus, early intervention could further train their fundamental language ability to proceed to the next step. This study, which demonstrated mature auditory cerebral function in preterm infants at their projected due dates, is a significant first step in understanding the cerebral mechanisms underlying language development in preterm infants. Furthermore, stuttering children and children with autistic spectrum disorders are reported as showing different lateralization patterns to the stimuli used in this study (Sato et al., 2011; Minagawa-Kawai et al., 2009). LI and HRF-typicality examined in this study could be used to assess language delay at certain points in the developmental process. Future study should further explore this possibility.

Although fNIRS has provided neurocognitive evidence in studies of basic neuroscience, several technical issues remain to be resolved. One such issue is the influence of systemic blood flow on fNIRS signals as reported by several studies (Kohno et al., 2007; Takahashi et al., 2011). On the other hand, a recent fNIRS-fMRI coregistration study reported the efficacy of fNIRS during a working-memory task (Sato et al., 2013). The study revealed that fNIRS-Hb significantly correlated with fMRI-BOLD rather than with the skin-blood flow in the adult prefrontal cortex. Further, on measuring Hb changes in both deep and shallow tissue layers in infants (Funane et al., 2014), cerebral rather than extracerebral hemodynamics greatly contributed to fNIRS signals during speech listening and in the resting state. Nonetheless, the systemic signal should always be carefully considered for fNIRS study, as systemic effects may have different effects depending on the nature of the cognitive task. Beyond systemic effects, there remain some issues in the clinical use of this technique. The study of fNIRS is a young, emerging field and therefore the neural substrates of the HRF pattern and its relation to other measures of physiological development are still unclear. Furthermore, the HRF state may differ depending on the state of wakefulness. These issues make it difficult to assess individual brain state precisely. Therefore, accumulation of basic data with fNIRS is still required for its clinical use.

Interpretation of the results of this study is limited by variation in the intrauterine environment of our participants. Intrauterine environment, reflected by ‘small for gestational age’, is known to affect long term outcomes of infants. Although we demonstrated a significant correlation between PMA and HRF-typicality after excluding birth weight, we did not exclude infants who were small for gestational age from our sample (Streimish et al., 2012). This limitation may explain why the correlation between PMA and HRF-typicality following partial correlation analysis controlling for GA was only marginally significant. Intrauterine environment may have acted as a cofounding factor in the partial analysis. However, a more appropriate interpretation of the marginal significance is that GA did impact on HRF-typicality in addition to PMA. Even with neural maturation as the infants get older, lower GA may still slow their brain development. Previous EEG studies on speech perception in preterm infants have consistently reported that brain maturation (equivalent to PMA) is more significant than duration of language exposure (PNA) (Pena et al., 2010; Bisiacchi et al., 2009; Pena et al., 2012). Our study examining both GA and chronological age may add to the evidence that GA rather than chronological age influence the cerebral basis of speech perception. This issue should be explored in future research.

Another limitation of this study is the lack of consideration of potential differences in the rate of complications and neurotoxic interventions in the participants. The risk of significant complications and neurotoxic interventions is higher for very premature infants than for late preterm infants, thus biasing GA and/or PMA quality. We also need to consider the effect of complications, including patent ductus arteriosus, BPD, and intraventricular hemorrhage. However, we excluded infants with complications, including congenital anomalies (e.g. congenital heart anomalies), moderate and severe BPD, grade 2–4 intraventricular hemorrhage, periventricular leukomalacia, NEC, and deafness. This is because we aimed to exclude risk factors other than premature birth itself that can influence hemodynamic regulations and/or cognitive development. Accordingly, we made it a criterion that ductus arteriosus was closed in all infants, and no participants received supplemental oxygen for BPD at the time of fNIRS measurement. Although we included 9 infants with moderate BPD (5 infants in the group of 33–35 weeks of PMA, 1 infant in the group of 36–38 weeks, 3 infants in the group of 39–41 weeks, and no infants in the term group, respectively), all infants were examined after the cessation of supplemental oxygen. Because the proportion of infants with mild BPD among groups was not significantly different (*p* = 0.116, Fisher's exact test), it is unlikely that inclusion of BPD had an effect on the hemodynamic response. Although grade I intraventricular hemorrhage was included for the participants' recruitment criteria, the final dataset of this study resulted in involving no patients with grade I intraventricular hemorrhage. Our exclusion criteria may have decreased the effect of such risk factors on our results. Many previous studies share similar limitations to those outlined here (Mwaniki et al., 2012; Luu et al., 2009; Aarnoudse-Moens et al., 2009; Bisiacchi et al., 2009). Likewise, the present study intended to examine the general tendency of preterm infants, rather than examining traits of any specific subgroup. The effects of specific traits—such as BPD and the maturity of cerebrovascular autoregulation, with regard to intraventricular hemorrhage—on the development of hemodynamic response discussed in this study should be further investigated in future research.

## 5 Conclusions

The present study has provided new evidence that hemodynamic regulation of the speech-evoked cerebral response and its functional systems develops in preterm infants as PMA increases and will typically catch up to term infants by their projected due dates. While an atypical HRF was frequently observed in these infants before 39 weeks of PMA, their HRF became matured by their projected due dates. Furthermore, functional hemispheric specialization of temporal regions responsible for prosodic processing became similar in preterm infants from 39 weeks of PMA, suggesting an efficient network for prosodic processing at this age. These data showing the maturation process of the hemodynamic response may represent a developmental milestone in higher brain functions, such as language processing, in preterm infants and may highlight the efficacy of early intervention for developmental cognitive delay. Further neurocognitive studies such as this can contribute to the understanding of physiological mechanisms of higher brain dysfunction in preterm infants.

The following is the supplementary data related to this article.

## Statement of financial support

This work was supported in part by the MEXT-supported program for strategic research foundations at private universities and MEXT KAKENHI; Grant numbers JP15H01691, JP24300105 (YM) and JP24591609, JP15K09725 (TA).

## Disclosure

The authors have no relevant financial relationships to disclose. The authors have no conflicts of interest to declare.
